# Anti-Inflammatory Activities of Leaf Oil from* Cinnamomum subavenium In Vitro* and* In Vivo*

**DOI:** 10.1155/2019/1823149

**Published:** 2019-02-20

**Authors:** Xincai Hao, Weiguang Sun, Changbin Ke, Fuqian Wang, Yongbo Xue, Zengwei Luo, Xuanbin Wang, Jinwen Zhang, Yonghui Zhang

**Affiliations:** ^1^College of Pharmacy Hubei University of Medicine, Shiyan 442000, China; ^2^Hubei Key Laboratory of Natural Medicinal Chemistry and Resource Evaluation, School of Pharmacy, Tongji Medical College, Huazhong University of Science and Technology, Wuhan 430030, China; ^3^Tongji Hospital Affiliated to Tongji Medical College, Huazhong University of Science and Technology, Wuhan 430030, China

## Abstract

The study determined the chemical constituents and anti-inflammatory effects of leaf oil from* Cinnamomum subavenium* (CS-LO) that has been used in folk medicine to treat various symptoms including inflammation. The anti-inflammatory effects of the oil were evaluated by LPS-stimulated RAW264.7 cells and the Carr-induced hind mouse paw edema model, respectively.* In vitro*, nitric oxide (NO), prostaglandin E2 (PGE_2_), TNF-*α*, IL-6, and IL-1*β* were significantly decreased by CS-LO, and the expression of nuclear factor-*κ*B (NF-*κ*B) protein was blocked as well. In* in vivo,* the malondialdehyde (MDA) and paw edema levels were decreased by CS-LO, and the same result came up on the NO and tumor necrosis factor (TNF-a) of serum at the 5th h after Carr injection. In addition, iNOS and COX-2 immunoreactive cells of the paw tissue were decreased significantly by CS-LO (200 mg/kg) in histological examination. The present findings indicated that CS-LO have anti-inflammatory properties, and the effects might be caused through inhibiting iNOS, COX-2, TNF-*α*, IL-1*β*, and IL-6 expression via affecting NF-*κ*B pathway, which will provide a power scientific basis for CS-LO to be used as the treatment of inflammatory diseases.

## 1. Introduction

The inflammation, associated with pain and swelling, is a series of the multiple biological reactions of body tissues to harmful stimulation. It is self-protective function of organisms to harmful stimuli [1]. Inflammation is used to be a hazardous factor for the onset of some chronic diseases, such as neurological disease, cardiovascular disease, and cancer [2]. Therefore, many studies considered anti-inflammation and antioxidant as a practical way to fight these degenerative diseases [3]. Plant extracts and essential oils, which are rich sources of antioxidant and anti-inflammatory agents, can suppress the release of inflammatory mediators and free radicals and increase antioxidant defenses. They are widely used in food, cosmetics, and medicine for their potential biological activities [4, 5]. Therefore, the use of natural additives is a good way to fight abnormal inflammation and oxidation.

The anti-inflammatory effect of some* Cinnamomum* oil has been confirmed, such as* Cinnamomum osmophloeum, Cinnamomum insularimontanum Hayata,* and* Cinnamomum cassia. *Much of the ingredients of the essential oils belong to terpenes, including monoterpene, sesquiterpene, and their oxidative derivatives. These small molecules tend to diffuse through the membranes to induce biological reactions.

*Cinnamomum subavenium.* Miq. belong to lauraceae family, which are popular in China, Malaysia, Cambodia, Indonesia, and Burma [6]. Its peel, fruit, and leaves have been used in folk medicine for treating stomachache, carcinomatous swelling, abdominal pain, chest pain, hernia, rheumatism, vomiting, nausea, and diarrhea [7]. Recently, researchers have reported that* C. subavenium *has potent cytotoxic effects in some tumor cell lines, including urothelial carcinoma cells, colorectal cancer cells, skin cancer melanoma cells, human bladder cancer cells, human lung cancer cell, and human prostate cancer cell lines [7–12]. Moreover, the leaf oil of* C. subavenium* has potent antioxidant and antimicrobial activities [13]. But, the anti-inflammatory activity of* C. subavenium* has not been report so far. Therefore, the objectives of our study were to investigate the major chemical composition of leaf oil from* C. subavenium* using gas chromatography-mass spectroscopy (GC-MS) and assess the anti-inflammatory activities of the leaf oil from* C. subavenium *(CS-LO).

## 2. Materials and Methods

### 2.1. Chemicals and Materials

Indo, LPS (*Escherichia coli* serotype 0111:B4), *λ*-carrageenan, and MTT (3-[4, 5-dimethylthiazol-2-yl]-2, 5-diphenyltetrazolium bromide) were obtained from Sigma-Aldrich Co. (USA). Prostaglandin E_2_ Express ELISA was from Cayman Chemical (USA). TNF-*α*, IL-1*β*, and IL-6 ELISA were from Boster Biological Engineering Co., Ltd. (China). Anti-iNOS, anti-COX-2 antibody, and anti-NF-*κ*B p65 antibody were from Santa Cruz Biotechnology (USA). BAY 11-7082 (NF-*κ*B inhibitor) was from Beyotime Institute of Biotechnology (China). The 4-amino-5-methylamino-29,79-difluorofluorescein diacetate (DAF-FM diacetate) was from Invitrogen (USA).

### 2.2. Plant Materials

Leaves of* C. subavenium *Miq were collected from China and identified by Changgong Zhang (Huazhong University of Science and Technology). The voucher specimen (No. 2012-0610) has been deposited at the Hubei Key Laboratory of Natural Medicinal Chemistry and Resource Evaluation, School of Pharmacy, Tongji Medical College, Huazhong University of Science and Technology.

### 2.3. Preparation of CS-LO

500 g fresh leaf of* C. subavenium* was hydrodistilled for 4 h with a Clevenger-type apparatus. The collected oil, dried with anhydrous sodium sulfate, was stored in brown vials at +4°C.

### 2.4. Gas Chromatography and Mass Spectrometry Analysis

The constituents of essential oil were identified by gas chromatography-mass spectrometry (GC-MS). MS is equipped with Polaris Q quality selective detector in electron collision ionization mode (70 eV). The chromatographic column was an RTx-5 (25 m x 0.25 mm x 0.25*μ*m), kept 1 minute at 80°C to 200°C at 4°C/min, and kept 5 minutes. The temperature of injector was 250°C, the helium gas flow rate was 10 mL/min, and the split ration was 1:10. Manual injection of diluted samples (1.0*μ*l, 1/100, v/v, ethyl acetate) was in nonshunt mode. The major chemicals of CS-LO were identified by comparison with standard products and retention indices based on their mass spectral fragmentation in The Wiley GC-MS library. The amount of compounds was determined by Peak area of integral spectrograms.

### 2.5. Cell Viability Assay

The RAW 264.7 cells were obtained from Cell Culture Center of Chinese Academy of Medical Sciences (Beijing, China). The cells were cultured in DMEM supplemented with FBS (10%), streptomycin (100 mg/Ml), and penicillin (100 U/mL) at 37°C in a humidified atmosphere with 5% CO_2_ and were subcultured every 3 days. The CS-LO was dissolved in DMSO and diluted with culture medium to target concentration. RAW 264.7 cells (1.5×10^5^) in 96-well plates were treated with 0, 2.5, 5, 10, 20, and 40 *μ*g/mL of CS-LO in LPS at 37°C for 24 h. Adding MTT solution to every well, the cells were incubated for another 4 h at 37°C. And then, the medium was discarded; the formazan crystals were dissolved in DMSO. The absorbance was measured at 570 nm.

### 2.6. Detection of Intracellular NO, PGE2, TNF-*α*, IL-1*β*, and IL-6

NO production was measured by the nitrite levels of the cultured media and serum based on the Griess reaction [14]. RAW 264.7 cells were incubated in 0.2 *μ*g/mL LPS with different CS-LO (2.5, 5, and 10 *μ*g/mL) for 18 h. Then, the collected medium was mixed with the same volume of Griess reagent (1% sulfanilamide and 0.1% naphthylethylenediamine dihydrochloride in 2.5% phosphoric acid). 15 min incubation later, the absorbance was detected at 540 nm using Micro-Reader. RAW 264.7 cells treated with LPS and CS-LO were incubated with DAF-FM (10 *µ*M) diacetate at 37°C for 1 h, and the production of NO was assessed by confocal laser scanning microscopy. The production of PGE_2_ was quantified with the Prostaglandin E2 Express EIA Monoclonal Kit. IL-1*β*, IL-6, and TNF-*α* were quantified with their ELISA kits under the manufacturer's instructions.

### 2.7. Western Blot Analysis

After washing with cold PBS, the stimulated RAW 264.7 cells were lysed in a cold lysis buffer [10% glycerol, 1% Triton X-100, 1 mM Na_3_VO_4_, 1 mM EGTA, 10 mM NaF, 1 mM Na_4_P_2_O_7_, 20 mM Tris buffer (pH 7.9), 100 mM b-glycerophosphate, 137 mM NaCl, 5 mM EDTA, and one protease inhibitor cocktail tablet (Roche, Indianapolis, IN, USA)] and incubated on ice for 0.5h. The collected supernatants were rapidly frozen. The concentration of the proteins was detected using the BCA method. After denaturation, the proteins of the cell extracts were separated by SDS-polyacrylamide gel electrophoresis. After electroblotted onto a PVDF membrane, they were incubated with blocking solution (5% skim milk) for 12 h at 4°C, and then incubated with primary antibody for 4 h. After washed with Tween 20/Tris-buffered saline (TBST), the blots were incubated with a dilution of horseradish peroxidase-conjugated secondary antibody for 1 h at room temperature. The blots were washed again with TBST and then developed using enhanced chemiluminescence. The quantities of western blot were made by measuring the relative intensity contrasted with the control using Kodak Molecular Imaging Software.

### 2.8. Carr-Induced Paw Edema

Male Wistar rats (180–220 g), two months old, were provided by the Hubei Provincial Center for Disease Control and Prevention. All animals were kept under 23–25°C conditions and 12 hours of light/12 hours dark cycles. The experiments and animal maintenance followed the animal care and use guidelines of the Institute's Animal Care and Use Committee of Huazhong University of Science and Technology. The Wistar rats, injected with 1% Carr (50 *μ*L) in the plantar side of right hind paws by 30min in advance, were administered with CS-LO (50, 100, and 200 mg/kg, p.o.), Indo (10 mg/kg, p.o.), and the vehicle (pure water + CMC 0.5%). The volume of paw was measured using a plethysmometer (model YLS-7B, Zhong Hao, Peking, China) before Carr injection and every hour after administration of the edematogenic agent for 5 h. The degree of edema (%) was measured by the ratio ((V1−V0)/V0), where V0 is the normal volume before injection, and V1 is the pathological volume after Carr injection. Finally, the rats were sacrificed; the collected blood and Carr-induced edema feet were stored at -80°C until analysis.

After being homogenized in PBS (pH 7.2) and centrifuged at 12000 × g for 5 min, the collected supernatants were stored at -20°C for MDA determination. The collected serum was stored at -20°C for the NO, PEG2, and TNF-*α* assay.

### 2.9. MDA Assay

MDA of the paw edema tissue was measured with the thiobarbituric acid reacting substance (TBARS) [15]. In brief, MDA was used to react with thiobarbituric acid to form a red-complex TBARS under high temperature and acidic conditions, and then it was determined at 532 nm.

### 2.10. Histological Examination

Histological examination was performed based on the methods by Huang et al. [16]. Briefly, after being dehydrated by ethanol, the tissue sections were paraffinized. After deparaffinization, some sections (thickness, 7*µ*m) were stained with hematoxylin and eosin (H&E); the others were processed for iNOS and COX-2 immunohistochemistry staining. BH2 Olympus microscopy was used to observe and photograph the samples. 3-5 tissue slices were randomly chosen from Control, Carr, Indo, and CS-LO-treated groups.

### 2.11. Statistics: Experimental

Data were presented as the mean ± standard deviation (SD) of three parallel measurements. The statistical significance was evaluated by one-way analysis of variance (ANOVA). Statistical significance is expressed as *∗*p < 0.05, *∗∗*p < 0.01, and *∗∗∗*p < 0.001.

## 3. Results

### 3.1. Chemical Composition of CS-LO

The essential oil was obtained from the leaves of* C. subavenium *by steam distillation and then analyzed by GC-MS. 39 compounds were identified in the CS-LO (Table 1). The main components included *β*-cadinene (12.45%), *α*-muurolene (8.79%), chavicol (6.46%), citral (5.33%), cis-*β*-ocimene (5.17%), *γ*-muurolene (4.34%), bornyl acetate (4.15%), *α*-copaene (3.78%), linalool (3.31%), methyl cinnamate (3.28%), geraniol (3.31%), and 3-allyl-6-methoxyphenol (3.17%). Among the constituents, sesquiterpenoids accounted for the highest fraction at 45.52% of the total, monoterpenoids accounted for 36.82%, and the nonterpenoids group accounted for 15.06%.

### 3.2. Effects of CS-LO on RAW 264.7 Cell Viability

**Figure 1:**
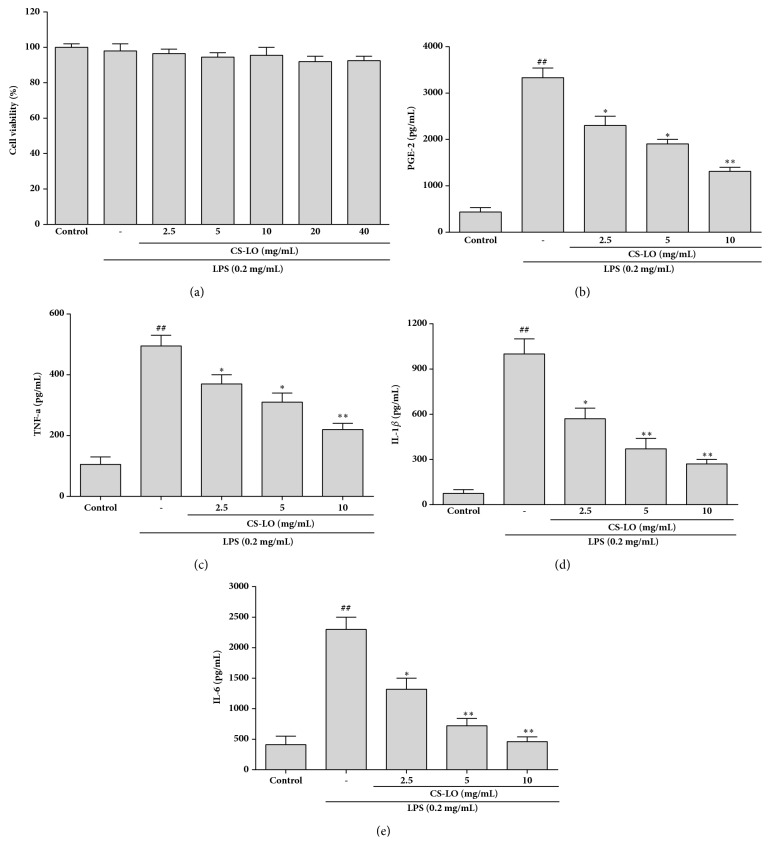
Effect of CS-LO on LPS-induced cell viability, PGE_2_, TNF-*α*, IL-1*β*, and IL-6 in RAW 264.7 cells: (a) cell viability, (b) PGE_2_ production, (c) TNF-*α* production, (d) IL-1*β* production, and (e) IL-6 production. The data represent the means ± SD values from three independent experiments. *∗* P < 0.05, *∗∗* P < 0.01, and *∗∗∗* and P < 0.001 compared to the LPS-treated cells alone.

The toxicity of CS-LO was determined in RAW 264.7 cells by MTT assay; the result is shown in Figure 1(a). Cell viability did not change at 0, 2.5, 5, 10, 20, and 40 *μ*g/mL of CS-LO. These results suggest that there were no toxic to RAW 264.7 cells under 40 *μ*g/mL of CS-LO. Therefore, CS-LO (2.5-10 *μ*g/mL) was used in the subsequent experiments.

### 3.3. Effects of CS-LO on LPS-Induced NO Production

**Figure 2:**
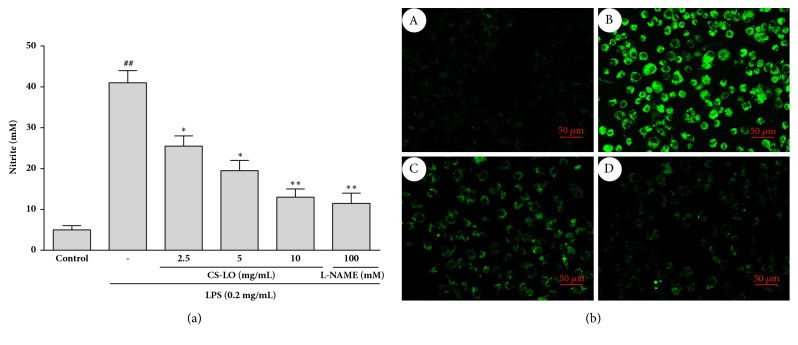
Effect of CS-LO on LPS-induced NO production. (a) The NO content was measured with Griess reagent. (b) The NO content was evaluated with DAF-FM diacetate by confocal laser scanning microscopy: A: cells alone; B: cells with LPS; C: cells with LPS and CS-LO; D: cells with LPS and L-NAME.

NO production may reflect inflammation; the effect of CS-LO on LPS-induced NO in RAW 264.7 cells was assessed. After treatment with CS-LO and L-NAME (NO inhibitor), the level of NO in RAW 264.7 was evaluated. As shown in Figures 2(a) and 2(b), there was a concentration-dependent inhibition in NO production. The group treated with 10 *μ*g/L CS-LO (p < 0.01) was highly decreased compared to the LPS-alone group, and L-NAME (100 *µ*M) also have a significant inhibition on the NO in RAW 264.7 cells (Figure 2(a)). Moreover, the image of confocal laser scanning microscopy indicates that the NO production was also suppressed by CS-LO (Figure 2(b)).

### 3.4. Effects of CS-LO on LPS-Induced PGE_*2*_ Production

PGE_2_ is another important mediator in inflammatory responses. Decreasing the PGE_2_ production would be an effective strategy for suppressing inflammation. As shown in Figure 1(b), PGE_2_ was inhibited by CS-LO. Especially, there was a significant inhibition on the expression of PGE_2_ at the 10 *µ*g/mL of CS-LO.

### 3.5. Effects of CS-LO on LPS-Induced TNF-*α*, IL-1*β*, and IL-6

TNF-*α*, IL-1*β*, and IL-6 are some important proinflammatory cytokines linked with the immunopathology inflammatory diseases. After stimulation with LPS (0.2 *µ*g/mL) and treatment with CS-LO, TNF-*α*, IL-1*β*, and IL-6 were detected by ELISA. As shown in Figures 1(c), 1(d), and 1(e), TNF-*α*, IL-1*β*, and IL-6 were upregulated after treated with the LPS. However, the inhibition of CS-LO on TNF-*α*, IL-1*β*, and IL-6 was in a dose-dependent manner.

### 3.6. Effects of CS-LO on NF- *κ*B Activity in RAW 264.7 Cells

**Figure 3:**
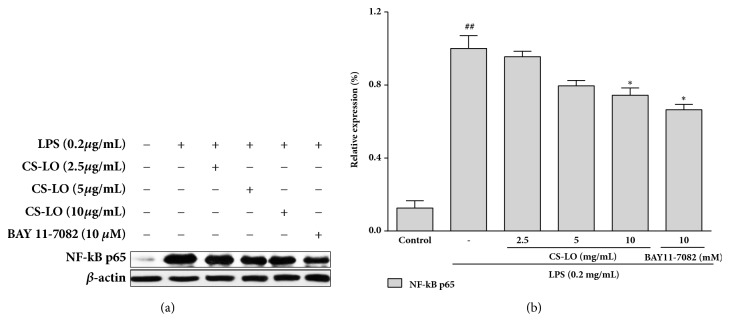
Effect of CS-LO on LPS-stimulated protein expression of NF-*κ*B p65 (a, b) in RAW 264.7 cells. The NF-*κ*B inhibitor BAY 11-7082 (10 *µ*M) was used as a positive control. *β*-actin was used as an internal control. The data are presented as the mean ± SD for three different experiments performed in triplicate. ## p < 0.01 as compared to the control group. *∗* P < 0.05, *∗∗* P < 0.01, and *∗∗∗* P < 0.001 compared to the LPS-treated cells alone.

The NF-*κ*B transcription factor plays a key role in the expression of some cytokines and inflammatory enzymes, such as iNOS, COX-2, TNF-*α*, IL-1*β*, and IL-6. To determine whether CS-LO affects NF-кB pathway, NF-кB p65, a major subunit of NF-кB, was assessed in the presence or absence of CS-LO using western blot. As shown in Figures 3(a) and 3(b), the expression of NF-кB p65 was increased significantly with absence of CS-LO in RAW 264.7 cells. However, the p65 (Figure 3(a)) was inhibited in concentration-dependent manner in cells with CS-LO.

### 3.7. Effects of CS-LO on Carr-Induced Rats Paw Edema

**Figure 4:**
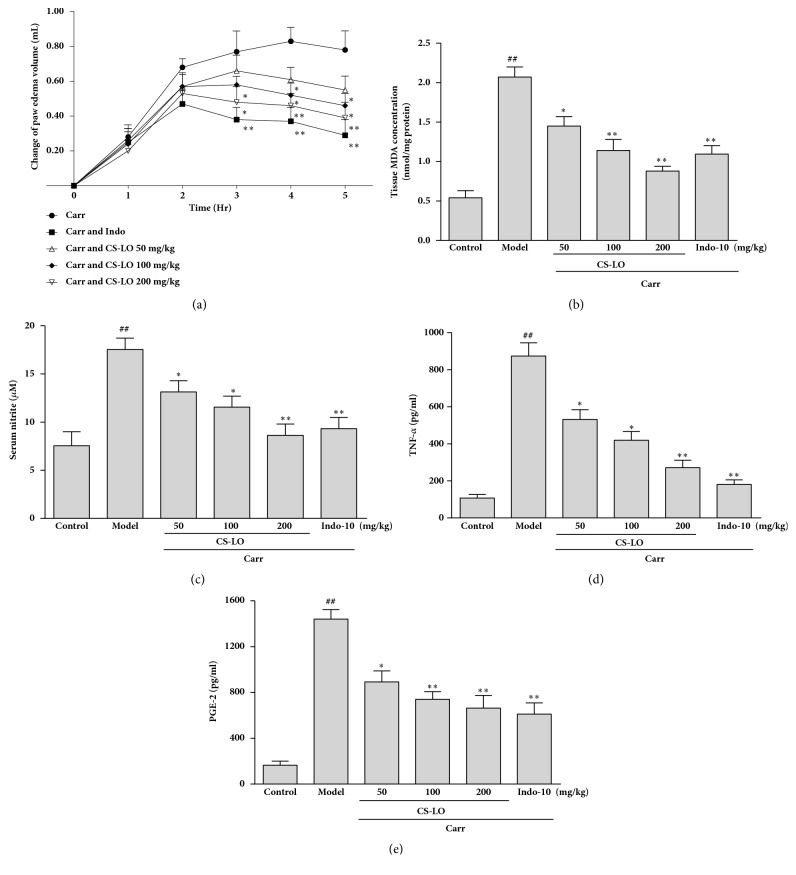
Effects of CS-LO on hind paw edema, MDA, NO, TNF-*α*, and PGE_2_ concentrations in rats. (a) Paw edema, (b) MDA of the tissue, (c) Carr-induced NO production, (d) Carr-induced MDA production, and (e) Carr-induced TNF-*α*production. The data are presented as the mean ± SD for three different experiments performed in triplicate. ### p < 0.001 compared to the control group. *∗* P < 0.05, *∗∗* P < 0.01, and *∗∗∗* P < 0.001 compared to the Carr group.

The effects of CS-LO on acute inflammation were tested with the Carr-induced paw edema model. As shown in Figure 4(a), there was a dose-related inhibition of CS-LO to hind paw edema. Especially at 200 mg/kg, CS-LO significantly inhibited (p < 0.01) the development of Carr-induced paw edema. Indo (10 mg/kg) significantly decreased the Carr-induced paw edema as well.

### 3.8. Effects of CS-LO on the MDA Level

MDA was measured to evaluate the ability of CS-LO to radicals. As shown in Figure 4(b), there was a higher level of MDA in paw tissue at 5 h after Carr injection (p < 0.01). However, dose-related inhibition of the MDA level was observed in paw tissue at 5 h after treatment with CS-LO (50, 100, and 200 mg/kg); CS-LO at 100 and 200 mg/kg significantly decreased the MDA level in the paw tissue (p < 0.01), the same with Indo (10 mg/kg) (Figure 4(b)).

### 3.9. Effects of CS-LO on the NO Level

As shown in Figure 4(c), at 5 h after treatment with Carr (p < 0.01), the NO level increased significantly in the serum of rats. However, the inhibition of CS-LO to NO in the serum of rats was found with a dose-dependent manner, and 200 mg/kg of CS-LO significantly reduced the NO level in the serum (p < 0.01).

### 3.10. Effects of CS-LO on TNF-*α* Level

As shown in Figure 4(d), the TNF-*α* level in the serum increased significantly at 5 h after treatment with Carr (p < 0.01). However, CS-LO highly reduced the TNF-*α* in the serum, and 200 mg/kg of CS-LO significantly reduced TNF-*α* level in the serum (p < 0.01).

### 3.11. Effect of CS-LO on PGE_*2*_ Level

As shown in Figure 4(e), the PGE_2_ level in the serum increased significantly at 5 h after treatment with Carr (p < 0.01). However, CS-LO decreased PGE_2_ in the serum with a dose-dependent manner, and CS-LO (100 and 200 mg/kg) highly inhibited the level of PGE_2_ (p < 0.01).

### 3.12. Histological Examination

**Figure 5:**
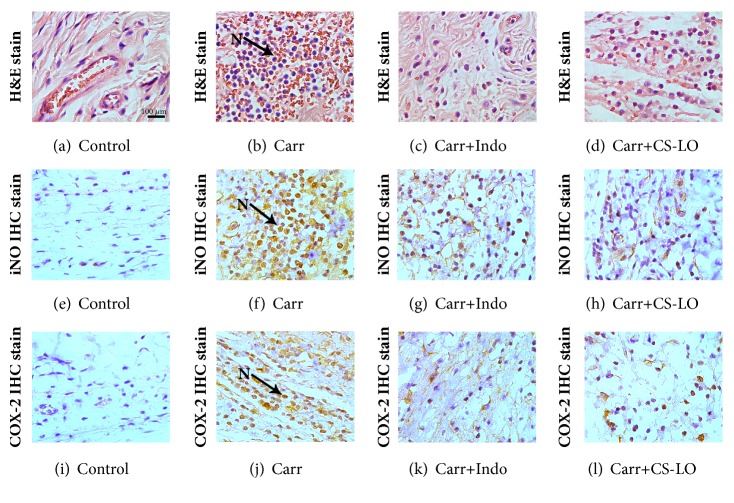
Histological observation of rat hind footpads after injecting Carr 0.9% saline (control group) or Carr. (a-d) H&E staining of footpad tissue sections from rat in each group. (e-h) iNOS immunohistochemical staining of footpad tissue sections from rat in each group. (i-l) COX-2 immunohistochemical staining of footpad tissue sections from rat in each group. Scale bar = 50 *µ*m. The infiltrating cells were predominantly neutrophils (N; arrows). The brown staining indicates the interaction of primary and secondary antibodies and the presence of iNOS and COX-2.

The histological appearance showed a normal appearance of tissue without any significant lesions in the control group (Figure 5(a)). Haemorrhage and a large number of neutrophils infiltrated the interstitial tissue in Carr-treated group (Figure 5(b)). CS-LO (200 mg/kg) and Indo reduced the number of neutrophils compared with the Carr-treated group (Figures 5(c) and 5(d)). There were no inflammation, iNOS, and COX-2 immunoreactive cells (Figures 5(e) and 5(i)); however, a large number of iNOS and COX-2 immunoreactive cells were found in brown parts of the plantar biopsy tissue at 5 h after injection of Carr (Figures 5(f) and 5(j)). Indo and CS-LO (200 mg/kg) significantly inhibited the iNOS and COX-2 immunoreactive cells in the paw tissue (Figures 5(g), 5(h), 5(k), and 5(l)).

## 4. Discussion

Inflammation is mediated by a series of free radicals and inflammatory cytokines. Excessive or persistent generation of these inflammatory mediators may cause diseases, such as arthritis, diabetes, atherosclerosis, and some types of cancer [17]. During inflammation, the activated macrophages are the source of inflammatory cytokines, which were important cells for phagocytosis and molecular immunology [18]. Moreover, many researches showed that the inflammatory free radicals could be induced by l-carrageenan (Carr), which was the suitable way to study the inflammatory effect.

Previous studies had demonstrated the bioactivity of the* C. subavenium*. For example, Wang et al. demonstrated the stems of* C. subavenium* had good pigmentation inhibitory abilities [12]. Cheng et al. found that the leaves and stems of* C. subavenium* had antioxidative activities [19]. Ho et al. reported that the leaf oils of* C. subavenium* had good antimicrobial activities [13]. Chen et al. reported the extraction of* C. subavenium* leaves had inhibition to various cancer cell lines [20]. So in this study, we analyzed the leaf oil of* C. subavenium* by GC-MS, and anti-inflammatory effects with LPS-induced RAW264.7 cells and Carr-induced paw edema in mice.

Chemical analysis revealed that there are 39 compounds in CS-LO, sesquiterpenoids account for the highest fraction of 45.52%, monoterpenoids account for 36.82%, and the nonterpenoids group account for 15.06%. Moreover, there was no dominant compound in CS-LO. Therefore, the biological activity may be due to the effects of minor ingredients or synergetic effects among the ingredients.

The proinflammatory cytokines NO and PGE_2_ plays important roles in inflammatory diseases. NO is an endogenous free radical substance synthesized from  _L_-arginine by nitric oxide synthase (NOS) in various animal cells and tissues [21]. PGE_2_ is mediated by cyclooxygenase (COX), which has COX-1 and COX-2 isoforms. Therefore, the inhibition of NO and PGE_2_ is an effective strategy for inhibiting inflammation [22]. Our result showed that the production of NO and PGE_2_ was significantly inhibited by CS-LO (Figures 2 and 1(b)); we also found that CS-LO downregulated iNOS and COX-2 with immunohistochemical staining (Figures 5(h) and 5(l)). Thus, the anti-inflammatory effect of CS-LO may due to its inhibition of NO and PGE_2_ via blocking the expression of iNOS and COX-2.

TNF-*α*, IL-6, and L-1*β*, a few of important proinflammatory mediators, play important roles in mediating and regulating inflammation* in vitro* and* in vivo*. TNF-*α*, one of the cytokines produced mainly by activated macrophages, can promote inflammatory activity by regulating some adhesion molecules [23]. IL-6 can promote the differentiation of T and B lymphocytes and the release of some inflammatory cytokines [24]. IL-1*β* was one of the earliest expressed proinflammatory cytokines [25]. Overexpression of both IL-6 and IL-1*β* is considered to be an important role in the pathophysiology of rheumatoid arthritis [26]. In this study, TNF-*α*, IL-1*β*, and IL-6 level, which were highly increased by LPS, were inhibited by CS-LO (Figures 1(c), 1(d), and 1(e)).

NF-*κ*B plays an important role in regulating immune system and inflammatory processes. Activated NF-*κ*B regulates the transcription of inflammatory cytokines and enzymes, such as iNOS, COX-2, TNF-*α*, IL-1*β*, and IL-6 [27]. NF-*κ*Bp65, a signal for NF-*κ*B activation, played an important role in the regulation of its transcriptional capacity. Once the NF-*κ*Bp65 is activated and phosphorylated, it could promote proinflammatory cytokines [28]. Therefore, NF-*κ*B signaling pathway is considered to be a therapeutic route against inflammatory diseases. Our findings suggest that CS-LO suppressed the nuclear translocation of p65 in a concentration-dependent manner (Figure 3). Therefore, CS-LO might regulate the transcription of COX-2, iNOS, and other inflammatory cytokines by antagonizing the transcriptional activity of NF-*κ*B.

Carr-induced paw edema could cause the peripheral release of inflammatory cytokines and mediators in tissue [29]; it is a common model to study inflammation. After administrating with Carr, the edema will rise to the high level, PGE2, TNF-*α*, and NO will be released during 1–5 h, and the malondialdehyde (MDA) will be produced by free radical attack on the membrane [30]. Edema and MAD are important criteria to evaluate anti-inflammatory activity of natural products [31]. Our results showed that CS-LO significantly decreased the paw edema at 5 h after treatment with Carr (Figure 4(a)), the MDA level was also significantly decreased (Figure 4(b)). These findings indicate that CS-LO has a potent anti-inflammatory activity* in vivo*. NO, PGE_2_, and TNF-*α* play an important role in inflammatory response [32]. Our results revealed that CS-LO decreased the NO, PGE_2_ and TNF-*α* level after treatment with Carr (Figures 4(c), 4(d), and 4(e)). We also found that CS-LO downregulated neutrophils with H*＆*E staining (Figure 5(d)). These results, along with the significant suppression of LPS-induced NO, PGE_2_ and TNF-a by CS-LO in macrophages, suggest that the anti-inflammatory effects of CS-LO might due to its suppression of NO, PGE_2_, and TNF-*α* synthesis in the peripheral tissues.

## 5. Conclusions

In summary, CS-LO showed potent anti-inflammatory properties. These activities may be due to the effects of minor components or synergetic effects of the components. The current study also demonstrated that CS-LO not only inhibited iNOS and COX-2 expression and the subsequent production of NO and PGE_2_ but also reduced the expression of IL-1*β*, IL-6, and TNF-*α in vitro* and* in vivo*. Moreover, the inhibition of CS-LO was related to inactivation of NF-*κ*B. The results of this study indicate that the leaf essential oil of* C. subavenium* is a potential anti-inflammatory agent. The Conclusions section should clearly explain the main findings and implications of the work, highlighting its importance and relevance.

## Figures and Tables

**Table 1:** Constituents of essential oil from leaf of *C. subavenium* by GC–MS.

Compound	RI^a^	Area (%)^b^	Method of identification
*α*-pinene	938	1.12	MS^c^, RI
camphene	952	1.13	MS, RI
*β*-pinene	979	0.97	MS, RI
*β*-myrcene	985	0.25	MS, RI, Co^d^
eucalyptol	1015	1.56	MS, RI, Co
cis-*β*-ocimene	1041	5.17	MS, RI, Co
*γ*-terpinen	1065	0.08	MS, RI, Co
linalool	1102	3.31	MS, RI
fenchol	1115	0.17	MS, RI
camphor	1120	0.38	MS, RI
borneol	1159	1.51	MS, RI
4-terpineol	1172	1.64	MS, RI, Co
*α*-terpinol	1191	2.37	MS, RI, Co
neral	1221	1.66	MS, RI, Co
geraniol	1228	3.20	MS, RI, Co
citral	1239	5.33	MS, RI
chavicol	1255	6.46	MS, RI
*β*-citronellol	1266	0.41	MS, RI
bornyl acetate	1268	4.15	MS, RI
*α*-cubebene	1345	1.66	MS, RI, Co
geranyl acetate	1356	2.41	MS, RI, Co
3-allyl-6-methoxyphenol	1359	3.17	MS, RI, Co
*α*-copaene	1366	3.78	MS, RI
cyclosativene	1369	0.43	MS, RI
longicyclene	1373	0.32	MS, RI
ylangene	1378	0.43	MS, RI, Co
methyl cinnamate	1380	3.28	MS, RI, Co
*β*-elemene	1392	2.47	MS, RI
*β*-caryophyllene	1415	2.82	MS, RI
*α*-gurgunene	1425	0.47	MS, RI
*β*-humulene	1460	1.65	MS, RI
*γ*-muurolene	1477	4.34	MS, RI
*α*-amorphene	1483	1.16	MS, RI
*γ*-himachalene	1485	1.96	MS, RI
*α*-muurolene	1488	8.79	MS, RI, Co
*β*-cadinene	1525	12.45	MS, RI, Co
1,4-cadinadiene	1536	1.45	MS, RI
patchouli alcohol	1659	1.34	MS, RI
benzyl benzoate	1760	2.15	MS, RI
Total		97.40	

^a^Retention index.

^b^Percentages were obtained by peak-area normalization on column DB-5.

^c^MS = mass fragmentation.

^d^Cochromatography with authentic sample.

## Data Availability

All the data used to support the finding of this study are included within the article.
